# A framework for significance analysis of gene expression data using dimension reduction methods

**DOI:** 10.1186/1471-2105-8-346

**Published:** 2007-09-18

**Authors:** Lars Gidskehaug, Endre Anderssen, Arnar Flatberg, Bjørn K Alsberg

**Affiliations:** 1 Chemometrics and Bioinformatics Group, Department of Chemistry, Norwegian University of Science and Technology, N-7491 Trondheim, Norway

## Abstract

**Background:**

The most popular methods for significance analysis on microarray data are well suited to find genes differentially expressed across predefined categories. However, identification of features that correlate with continuous dependent variables is more difficult using these methods, and long lists of significant genes returned are not easily probed for co-regulations and dependencies. Dimension reduction methods are much used in the microarray literature for classification or for obtaining low-dimensional representations of data sets. These methods have an additional interpretation strength that is often not fully exploited when expression data are analysed. In addition, significance analysis may be performed directly on the model parameters to find genes that are important for any number of categorical or continuous responses. We introduce a general scheme for analysis of expression data that combines significance testing with the interpretative advantages of the dimension reduction methods. This approach is applicable both for explorative analysis and for classification and regression problems.

**Results:**

Three public data sets are analysed. One is used for classification, one contains spiked-in transcripts of known concentrations, and one represents a regression problem with several measured responses. Model-based significance analysis is performed using a modified version of Hotelling's *T*^2^-test, and a false discovery rate significance level is estimated by resampling. Our results show that underlying biological phenomena and unknown relationships in the data can be detected by a simple visual interpretation of the model parameters. It is also found that measured phenotypic responses may model the expression data more accurately than if the design-parameters are used as input. For the classification data, our method finds much the same genes as the standard methods, in addition to some extra which are shown to be biologically relevant. The list of spiked-in genes is also reproduced with high accuracy.

**Conclusion:**

The dimension reduction methods are versatile tools that may also be used for significance testing. Visual inspection of model components is useful for interpretation, and the methodology is the same whether the goal is classification, prediction of responses, feature selection or exploration of a data set. The presented framework is conceptually and algorithmically simple, and a Matlab toolbox (Mathworks Inc, USA) is supplemented.

## Background

An important part of expression data analysis is the significance analysis of genes, in which relevant features are found among thousands of irrelevant ones. Two widely used algorithms for significance analysis are SAM [1] and Limma [2]. These methods are well suited for assessing differences of expression across classes or levels in an experimental design. Dimension reduction methods such as principal component analysis (PCA) and partial least squares regression (PLSR) [3-5] is another group of popular tools for expression data analysis. These methods attempt to describe the underlying variation of the data in a low-dimensional space, enabling the use of statistical methods that cannot deal with the high number of correlated gene-expressions in the original data. The real signal is separated from random noise, and the components of the dimension reduction can often be interpreted biologically.

A much used dimension reduction method is PCA [3,4]. This is an unsupervised method that describes the main features of a data set using a small number of orthogonal components. The computational method of singular value decomposition (SVD) is in many aspects the same as PCA, and often it constitutes the main part of the PCA algorithm. When background knowledge is available about the objects, supervised methods may be used to identify genes that associate with this information. For instance, t-tests may be used to discriminate between classes in an experimental design. Also, correlation tests such as Spearman's correlation may be used to probe expression profiles for similarity with continuous responses. PLSR [3,5] is a very powerful dimension reduction method which can utilise any sort of background information directly in the decomposition. PLSR components are designed to optimally describe the dependent variables in terms of the expression data. The main focus of PLSR in the microarray literature has been on classification, where a regression model is trained to fit a binary array representing class labels. Many methods are available to convert the prediction model to a classification rule, for instance by discriminant analysis of the PLSR components [6,7]. We use the intuitively pleasing property that the predicted, validated responses may be discretised directly in order to predict class-assignments. When PLSR is used for classification it is referred to as discriminant PLSR (DPLSR) or PLS discriminant analysis (PLS-DA). Nguyen and Rocke were the first to demonstrate how DPLSR may be applied to classify cancers based on expression data [6,7]. In that work, they used an additional discriminant analysis for the final classification. PLSR has also successfully been used to model patient survival times [8] or cell cycle data [9], which are applications where a continuous response is used directly as input. Overviews of dimension reduction methods for analysis of microarray data are given in [10,11].

The dimension reduction methods offer a new way of doing significance analysis on expression data. The underlying variation of interest is described by a limited number of components, also known as latent variables (LVs). The LVs are axes in a coordinate system that span, depending on the dimension reduction method used, the most relevant structures in the data set. Each object (array) is associated with a limited number of scores, which represent the projection of the measurements onto the latent axes. There is also a loading vector for each component, providing a mapping between the latent and the original variables (features). The score vectors thus span interesting directions in the original feature space, and the accompanying loading vectors hold the contribution of the features in those directions. Jack-knife is a method which can be used to assess the significance of variables from the stability of cross-validated model parameters [3,12]. Genes important for class separation may be found by jack-knife of the components that best span the different classes. We use a modified version of Hotelling's *T*^2 ^test statistic [13] that is specially tailored for cross-validated data. The framework presented is a very general tool for significance testing, and it is applicable in principle to any dimension reduction method from which cross-validated loadings can be obtained. Once a dimension reduction method is chosen based on the data available or the experimental setup, the same methodology for significance analysis may be used whether one, several or no responses are included in the modelling. The dependent variables may describe continuous phenotypic information, categorical information or a combination of both.

We apply our framework for significance analysis on three publicly available expression data sets, including one with known spiked-in transcripts. Differences between PCA and PLSR-methods will be explored, and the results will be compared to SAM and Limma where possible. For ordinary PLSR or DPLSR, some of the weaker correlation patterns between the expression data and the responses may be overlooked in the modelling. We have therefore chosen to use a variant of PLSR called Bridge-PLSR [14]. This method is able to extract all information relevant to the responses in the first components, and is therefore better suited for this kind of significance analysis. The false discovery rate (FDR) is estimated by resampling to provide a threshold for significance [9,15]. The set of parameters obtained from either of the dimension reduction methods in this work is referred to as a bilinear model. This reflects that the components representing the data are linear in both the rows and the columns. The same term is used also about the mathematical formulation of how the data are represented by scores and loadings, but the exact meaning in each case should be clear from the context. Bold uppercase letters denote matrices, and bold lowercase letters describe column-vectors. An expression data set is arranged in an array **X **with experiments along the rows and with a column for each feature.

A toolbox written in Matlab (Mathworks Inc. USA) with the code used in this work is provided under the GNU general public license (see additional file 1).

## Results

### Smoker transcriptome

**Figure 1:**
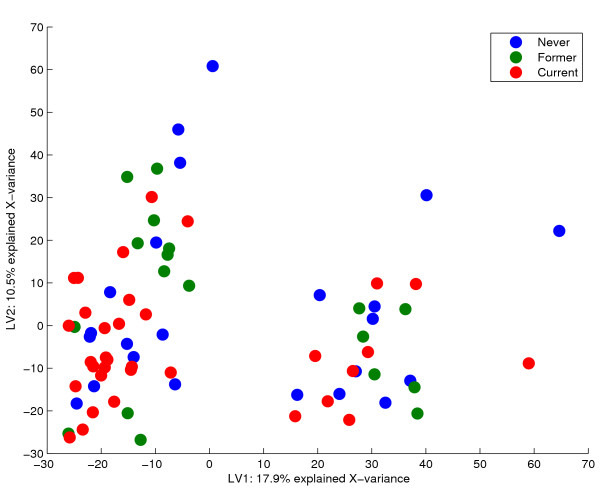
PCA scores. The scores from the PCA of the smoker-data plotted for the two first components. There is a major source of variation along the first component that does not correspond to the smoking history of the test subjects. These components are only able to explain 1% of the variance in Y.

**Figure 2:**
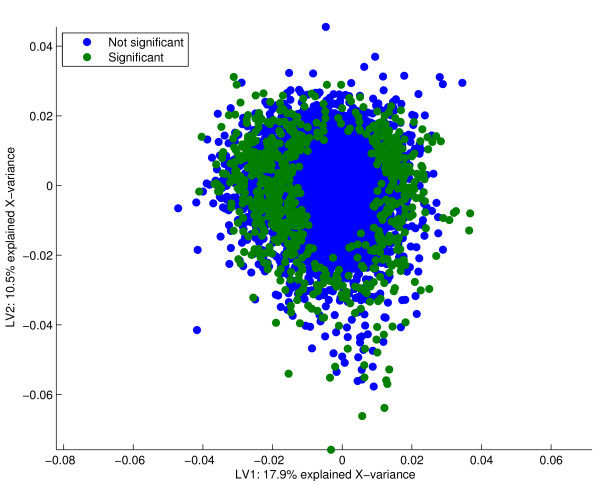
PCA loadings. The loadings from the PCA with the 725 most significant genes given in green. The actual number of significant genes is arbitrary, and corresponds to the number estimated from resampling of a Bridge-PLSR model. It is seen that the significant genes are scattered outside an elliptic shape centred at the origin. Genes with loadings of a large magnitude that vary little in the cross-validation are called significant. As neither of the components span the smoking history of the subjects, these features are irrelevant for classification.

**Figure 3:**
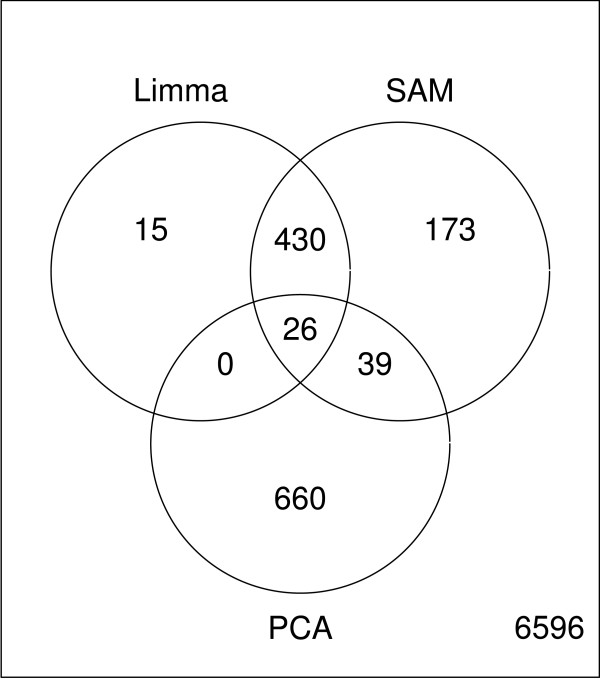
Significant outcomes from PCA. Venn diagram comparing the significant genes from PCA with SAM and Limma. The overlap between the supervised methods and the unsupervised PCA is very small for this data set. This is expected as the principal components do not span the groups of different smoking history.

We first look at a data set that investigates the effects of smoking on human airway transcripts [15]. Data are available from three groups of subjects according to their smoking history. The original article reports significant differences both between a group of smokers compared to subjects that have never been smoking, and between former smokers and the group of never smokers. A PCA of the expression data **X **reveals that there is a large source of variation in the data that does not correspond to the available group information. In figure 1, a PCA score plot of the two first components is given, where the objects are coloured according to the classes "Never", "Former", and "Current". Unreported experimental factors, for instance a change in laboratory procedures, may be responsible for the separation of objects into two clusters along the first component. In the corresponding loading plot in figure 2, the 725 most significant genes from the PCA analysis are given in green. These genes span mainly the unreported **X**-variation and are not important for the smoking-induced differences of expression. The Venn diagram in figure 3 confirms that the genes detected by PCA are to a large degree irrelevant for classification of these data.

**Figure 4:**
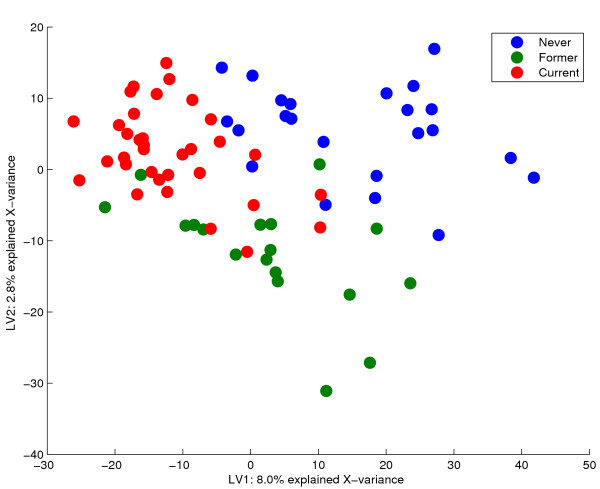
Bridge-PLSR scores. The two first Bridge-PLSR score-vectors describing the smoker-data are plotted. These components account for 54% of the (calibrated) variance of Y, but only 11% of the X-variance is explained by the model. The first component distinguishes well between current and never smokers, while the second component spans the minute variation that separates former smokers from the rest.

**Figure 5:**
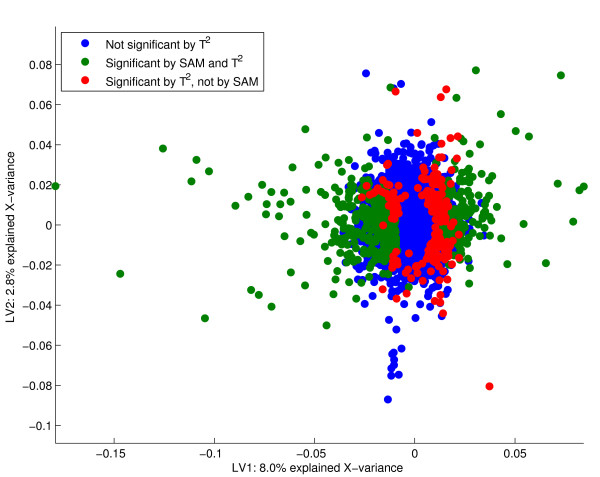
Bridge-PLSR loadings. Loadings from the Bridge-PLSR of the smoker-data are plotted for the two first components. The blue spots represent features that are not found significant by the jack-knife procedure. The green spots are genes that are found significant by both SAM and Bridge-PLSR, while the red spots are called significant by the *T*^2^-test but not by SAM. The significant features span mainly the direction of smokers vs. never smokers, but Bridge-PLSR detects some genes relevant for former smokers as well.

The PCA-analysis indicates that the group information should be allowed to guide the decomposition if any genes related to smoking are to be found. We know that a minimum of *K *- 1 independent variables are needed for a linear separation of *K *classes. For instance, one gene measured for several subjects may in theory be used to differentiate between two classes, while a minimum of two genes are needed for assignments into three classes. Due to co-regulation and inter-dependencies between genes, such single, descriptive features are usually both uninformative and hard to find. However, dimension reduction offers independent linear combinations of genes that may be used instead. If **Y **is a binary matrix holding the class information for each object, a good linear separation based on the expression data may be obtained with components that span all the **Y**-related variance in **X**. In Bridge-PLSR, this variance may be completely described in a minimum number of components. The score plot from a two-component Bridge-PLSR model is given in figure 4. Here, three groups corresponding to the predefined classes are revealed. This model explains 54% of **Y**, whereas an ordinary PLSR for comparison explains 45% in two components. Jack-knife is performed based on a full leave-one-out cross-validation, and the significant features are given in the loading plot in figure 5. The green spots correspond to features called significant by both SAM and Bridge-PLSR, whereas the red spots indicate significant genes not found by SAM. Neighbouring genes in the loading plot have similar profiles, whereas genes in opposite quadrants are negatively correlated. Also, the arrays in a specific area of the score plot have a high expression value for the genes in the corresponding area of the loading plot. As the significant genes span mainly the first component, they discriminate well between smokers and never-smokers, according to the score plot. Along the second component, which separates former smokers from the rest, fewer significant genes are found. This indicates that this correlation is weaker and more susceptible to random variations in the data. It is seen, however, that Bridge-PLSR finds more genes than SAM along this direction. Any significant genes along the second component describes smoking-induced transcriptomic changes among the former smokers.

**Figure 6:**
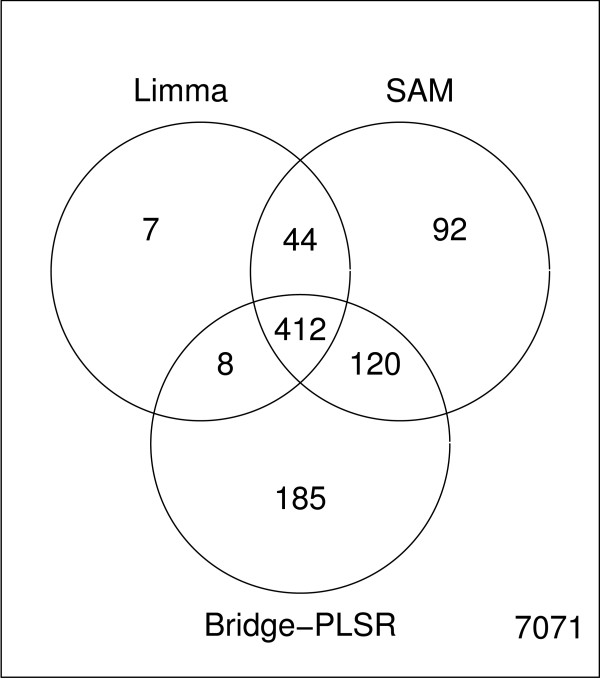
Significant outcomes from Bridge-PLSR. A venn diagram comparing the significant genes from Bridge-PLSR with SAM and Limma. At a significance level of 5%, the *T*^2^-test finds 725 significant genes, while SAM and Limma find a total of 668 and 471 features, respectively. The majority of the genes are found by all three methods.

A Venn diagram which compares the significant genes found with Bridge-PLSR, SAM and Limma is given in figure 6. All methods agree on the significance calls for the majority of the genes, however, Bridge-PLSR finds an additional 185 genes which are undetected by the other methods. To verify that these genes are not primarily false positives, the genes are annotated and their biological significance with regard to smoking is evaluated. A comprehensive list of our findings is given as supplementary information (see additional file 2). Of the genes identified as differentially expressed by Bridge-PLSR, only 66 have unknown or poorly known biological function. An additional 14 have well understood biological function often related to the natural system, but with no readily apparent link to smoking or lung damage. Of the remaining genes, 49 are known to be involved in regulation of cell growth, cell cycle, or apoptosis, processes known to be affected by smoking [16,17]. Seven of these genes as well as another 14 genes on the list not related to cell growth, have been shown to be involved in various forms of lung cancer. As the majority of lung cancer sufferers are smokers or previous smokers, these genes can be linked to smoking directly. Another interpretation is that these genes change early in the cancer development process. Seven genes are identified that have been reported to take part in lung development, this is not surprising as smoking can cause dramatic changes in the airways. In addition to cancer, we identify 17 genes that are related to other diseases in the lungs and bronchia, such as asthma, inflammation, fibrosis, or response to metallic toxins. Another large group of genes belongs to the protein life cycle, particularly the ribosome, or the ubiquitin cycle. This could point to an overall change in the rate of protein turnover caused by smoking. This is consistent with the results of Tomfohr *et al. *[18], who analysed the same data set with gene set enrichment analysis. Five genes are also found that are known to be expressed in lungs, but for which we find no link to cancer development or lung damage in the literature. Overall it seems that of the genes for which biological background information is available, the majority relate to biological processes that are influenced by smoking. The additional genes found by Bridge-PLSR are therefore unlikely to be false discoveries.

For prediction purposes, ordinary PLSR seems to be superior to Bridge-PLSR. The best predictive PLSR model is obtained for two components, with an explained **Y**-variance of 32%. The best predictive Bridge-PLSR model is a one-component solution with a cross-validated explained variance in **Y **of 20%. The residual mean squared error of prediction even increases slightly when two components are used. Caution must therefore be used when interpreting the second component. They are both included here because the main goal of the analysis is significance testing. Hotelling's *T*^2^-test using only one component corresponds to an ordinary one-sample *t*-test, and is, in this case, only able to separate between the groups "Never" and "Current". In order to find genes that might be relevant for former smokers, the second component must be investigated. The jack-knife is believed to reject most of the genes that cause the residual error to increase, as those genes have a high variance across cross-validation segments. For prediction purposes, the model with the lowest residual error might be used instead. It should be noted, however, that even if the Bridge-PLSR or PLSR-model with one or two components, respectively, is optimal for prediction, neither will be able to predict former smokers.

A ridge parameter *γ *controls the influence of **Y **in the decomposition. A small value of this parameter ensures that the first Bridge-PLSR components span the directions of maximum covariance between **X **and **Y**. This is ideal from the point of view of significance analysis, as the major components will span all the **Y**-relevant variance. However, if *γ *= 0, the maximum number of components is limited by the rank of **Y**, which for these particular data is two. For explorative reasons, *γ *is set to a default value of 0.01 throughout this work in order to enable interpretation also of later components. For the first two components, this has no visible effect compared to a *γ*-value of zero. Components three and four, however, are almost identical to the first two PCA components in figure 1 (not shown). This is a further verification that the Bridge-PLSR is able to separate the **Y**-relevant structures in **X **from technical and other irrelevant variation. It is possible to optimise both the ridge parameter and the number of components to minimise the residual error of the bilinear model. This would require an extra layer of validation, for instance by cross model validation [19], to obtain unbiased error estimates. As the primary goal in this work is significance analysis, such an optimisation is out of scope. However, a supplementary response surface is provided for reference, along with the ordinary PLSR results (see additional file 3). It can be seen that the cross-validated error variance is robust with regard to *γ *for small values of the parameter.

Analysis by ordinary PLSR is performed for the sake of comparison. This model uses two components to separate never and current smokers, and additional two or three components are needed to describe the former smokers. The optimal number of components according to the cross-validation is two, but the majority of the significant genes found by this model span only the irrelevant **X**-information (not shown). Because of the irrelevant, dominating structure in **X**, Bridge-PLSR is a much better choice for significance analysis in this case.

### Spiked-in data

A further analysis is performed with Affymetrix spike-in data in a constant human background [20]. The spiked-in transcripts hybridise to 42 known probe sets, however, several authors report that also other probe sets show differential expression due to sequence or function similarity. Sheffler *et al. *[21] remove 56 such genes as outliers prior to analysis. Two independent groups [22,23] suggest that additional 22 or 23 probe sets, respectively, should be added to the original list of known spike-ins. These lists differ in a single probe set, which is known to have identical sequence to one of the spiked-in genes for 5 of 11 probe pairs. We include the 22 extra genes which are suggested by both groups in our analysis, yielding a total of 64 spike-in probe sets. Bridge-PLSR with jack-knife finds all these genes among the 66 most significant ones. An estimated FDR of 0.01 results in 69 significant genes, corresponding to a true FDR of 7%. The five false positives are the probe sets "203173_s_at", "204890_s_at", "204891_s_at", "217019_at", and "200822_x_at". The maximum squared correlation coefficients between the expression values of these probe sets and the concentration profiles are, respectively, *r*^2 ^∈ {0.92, 0.88, 0.86, 0.33, 0.29}. For comparison, *r*^2 ^for the list of true positives range from 0.39 to 0.74. In passing, it is noted that the three false positives with high correlation are included also in the outlier-list of Sheffer *et al. *[21].

### Rat liver study

A data set investigating liver damage in rats after exposure to toxic doses of paracetamol is also analysed [24]. In addition to design information of dose and time of exposure, several pathological variables based on blood and tissue chemistry are available for the rats examined. There is an abundance of possible comparisons in these data, and analysis by Limma or SAM is therefore difficult. We perform an analysis by SAM in which two groups of "high" and "low" dosage are defined for each of the four time-points of the design. The drastic treatment some of these rats receive induces profound genetic responses, leading to a long list of differentially expressed features. Testing for all 28 contrasts defined by the 8 groups, SAM assigns significance to 50% of the genes at a significance level of 0.001. This result verifies something that might be expected – that poisoning of an organism can affect several biological processes that in turn involve many genes. Investigation of more complex relationships based on these methods is not straightforward.

**Figure 7:**
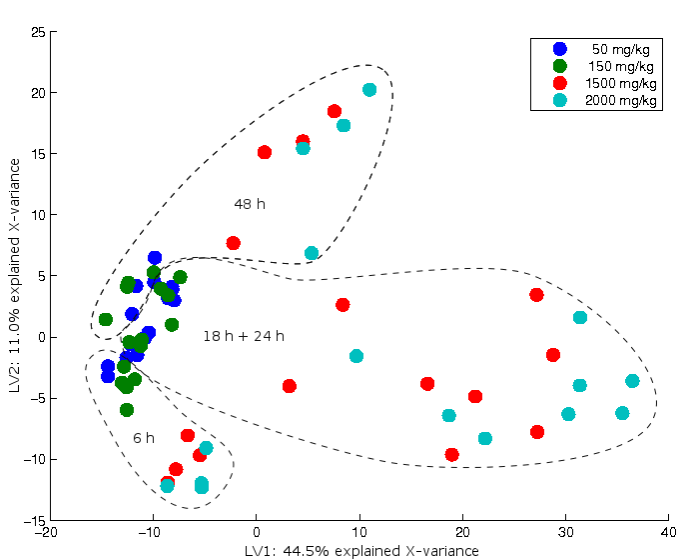
Bridge-PLSR scores. The score plot for a two-component Bridge-PLSR model of the rat-liver data. Each array is represented by a spot coloured according to the administered drug-dose. The dashed lines indicate the location of design-points corresponding to different times of exposure. Two groups of high and low dosage can be seen along the first component, and the second component seems to model a time-effect. The two-component model accounts for 47% of the total variance in Y.

PLSR is well suited for handling both multiple comparisons and continuous responses. The score plot from a Bridge-PLSR model with all available response information in **Y **is given in figure 7. The spots are coloured according to the administered drug-doses. The low doses are clearly separated from the toxic doses along the first component, but the classes within those two groups are mixed together. The dashed lines envelope three groups that are related to the time from drug exposure to necropsy. The lower group consists of the 6-hour design points, the upper group corresponds to the 48-hour points, while in the middle group the 18- and 24-hour points are mingled together. The second component is thus related to time since exposure. A leave-one-out cross-validation is performed also for these data. The optimal model for prediction is obtained after four components, with a residual variance of 47%. However, we are interested mainly in the genes which are differentially expressed across dose and time. We therefore use a two-component model in the significance analysis, with a prediction error of 58%. For these data with much structure in **Y**, the differences between the results from Bridge-PLSR and ordinary PLSR are small. All predictive components describe the same phenomena, and the residual errors are similar, both in a calibrated and validated sense. For reference, the full response surface including several values *γ *of is given as supplementary information (see additional file 3).

**Figure 8:**
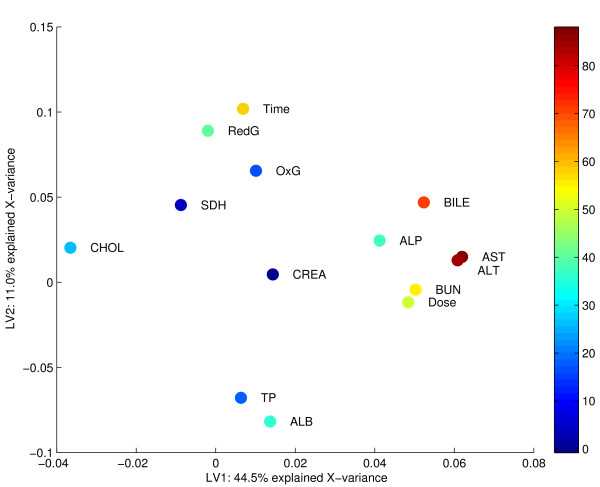
Bridge-PLSR Y-loadings. Bridge-PLSR Y-loadings for a two-component model of the rat-liver data. Each point represents an Y-variable, and the percent explained, validated variance for each response is indicated by the colour-bar on the right. The variables are the design parameters Dose and Time, the concentrations of reduced (RedG) and oxidised (OxG) glutathione levels in the liver, and the concentrations in the blood of sorbitol dehydrogenase (SDH), total bile acids (BILE), alkaline phosphatase (ALP), aspartate aminotransferase serum (AST), alanine aminotransferase (ALT), total protein (TP), albumin (ALB), blood urea nitrogen (BUN), creatinine (CREA) and cholesterol (CHOL). The diagnostic markers for liver injury ALT and AST is highly predictive for the first component, while the second component is related to time since exposure.

A helpful plot is the **Y**-loading plot in figure 8. Here, each spot corresponds to a response-variable, and the predictabilities are given by the associated colour-bar. The colour of a spot reflects the cross-validated, explained variance for that variable, given in percent of the true response's variation. The variables with red spots are very well predicted while the blue spots represent variables that cannot reliably be modelled based on the expression data. Two variables which have high predictive abilities and are correlated with the dose are ALT (alanine aminotransferase) and AST (aspartate aminotransferase). Increased levels of these enzymes in the blood stream are diagnostic markers for liver damage. The large degree of explained variance for these variables indicates that the first component actually describes liver injury directly, rather than just the concentration of paracetamol in the rats. Because the individual responses to the drug vary, these markers are better suited to model the genetic response to overdose than the design is. This information should be included also in the interpretation of the scores in figure 7. It is seen that the response to overdose peaks between 18 and 24 hours after exposure, as these groups span the first component better than the 6- and 48-hour groups. After 48 hours, metabolism may have disposed of some of the paracetamol, the liver-cells may have started to repair, and the levels of ALT and AST in the blood have started to decrease. Other correlations seen in figure 8 may also be of interest. Liver damage seems to be associated with increased concentrations of total bile acids and blood urea nitrogen, and there is also an indication that the level of cholesterol decreases. The reduced glutathione level in the liver tissue increases with time after poisoning, and the level of blood albumin seems to decrease accordingly.

**Figure 9:**
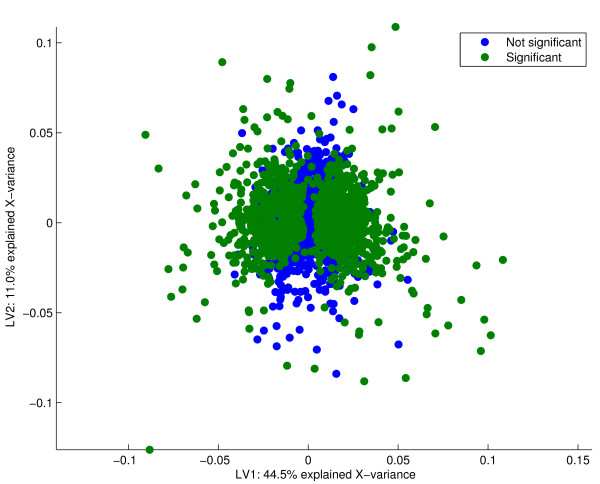
Bridge-PLSR X-loadings. Bridge-PLSR X-loadings for the rat-liver data. Many significant features are found, and most of them are related to liver damage as described by the first component. Some significant genes are also found that span the time of exposure.

An estimated FDR significance cutoff of 0.1% yields 25% significant features for the Bridge-PLSR model. The **X**-loading plot is given in figure 9, with the significant genes marked by green spots. The majority of these features span the component describing liver injury, but some are also found that vary with time. The significant genes found by SAM are scattered more uniformly over the same loadings, making any systematic trends hard to detect (not shown).

## Discussion

Dimension reduction methods are explorative in nature, and the decomposition attempts to find components that describe as much structure as possible using a small number of components. This is a very effective way to detect the sources of variation in large data tables, and visual inspection of score and loading plots enables discovery of unknown phenomena. The information carried by the components often corresponds to underlying biological processes, greatly simplifying interpretation of the results. Comparison of score and loading plots forms a central interpretative tool for mapping measured genetic responses to different subjects or levels of an experimental design. The dimension reduction methods may be vulnerable to outliers and systematic noise, and it is important that the results are properly validated and understood. The inspection of scores and loadings can be used as an aid in the discovery of bad arrays or bad spots, respectively.

Most genes are known to interact with other genes in potentially complex cellular networks. Investigation of one gene at a time is therefore not optimal for a good understanding of a biological system. The dimension reduction methods attempt to find linear combinations of genes that describe the data well, and are therefore better equipped to model co-regulated genes. The representation of expression data by a small number of components also enables use of statistical methods that assume independence. The significance testing itself is also multivariate, as features are tested for significance in multiple dimensions simultaneously. Genes that are not significant along any of the individual components may still be important for the full model.

The dimension reduction methods are applicable to a wide range of problems. PCA is widely used for clustering and for exploration of the main structure of a data set. In this work, PCA revealed a large systematic variance in the smoker data which was irrelevant for classification. A visual inspection of the scores clearly showed that a supervised decomposition was needed. PLSR has been much used in the classification of expression data, and other uses have been shown where the **Y**-information is non-categorical. It is hard to control all levels of variability in living organisms, and the responses to stimuli may differ between test subjects. Similarly, biopsies used in cancer classification are heterogeneous material and may vary even among patients with the same diagnosis. The direct use of clinical and pathological parameters in the analysis may enable a more accurate modelling than if just the classes or levels of a design are used.

While significance analysis by jack-knife is much used in other fields [3], at least two new concepts are introduced in this work. One is to use Bridge-PLSR to describe more of the response variation in fewer components. This may be needed when there is a large number of noisy or irrelevant variables in **X**, which is typical for expression data analysis. For the rat-liver data, no discernible difference was found between Bridge-PLSR and standard PLSR. For the smoker data, PLSR was more predictive for future objects, however, Bridge-PLSR was able to describe the classes better and is preferred for significance analysis. It is acknowledged also in [25] that the best genes for prediction are not always the most interesting ones. The second novel concept is the modified Hotelling's *T*^2^-test for use with jack-knifed models. This test is believed to be superior to the standard alternative of performing a t-test for each component individually.

There are several possible extensions to the presented framework that may be included to handle more complex problems. For instance, dimension reduction algorithms exist that inherently handle missing data [4]. Often background information about the features are available, for instance in the form of gene ontology or metabolic networks [26,27]. There are various ways to include *a priori *information in the analysis, either in the form of a pre-processing step or in a specialised algorithm [28]. The dimension reduction methods are not even restricted to two-way data. It is possible that a three-way organisation of the rat-liver data into an array of time-by-dose for each gene would result in a more stable decomposition. Several possible dimension reduction methods exist also for arrays of higher order [29,30]. Finally, the presented methodology is not restricted to expression data. Any bioinformatics application producing many measured variables per available object can be expected to benefit from these methods.

## Conclusion

The dimension reduction methods are multivariate and well suited to deal with the intricate network of dependencies between genes. They offer a versatility that allows any continuous or discrete background information available to be incorporated in the analysis. The results are intuitively understood based on simple scatter plots, which also enable the discovery of unknown phenomena in the data. The identification of differentially expressed genes is an important part of expression data analysis. We have presented a general scheme for significance testing that can be applied to a wide range of dimension reduction methods. The validity of the procedure has been established by comparison with other, well known algorithms for significance analysis, by biological interpretation, and by analysis of spike-in data.

## Methods

### Data sets

The smoker data [15] contain expression levels for *M *= 7939 probe sets measured for *N *= 75 subjects on Affymetrix HG-U133A GeneChips (Affymetrix, Inc., USA). The subjects are divided into 34 current smokers, 18 former smokers and 23 individuals that have never been smoking. The expression data are arranged in a matrix **X **of size *N *rows by *M *columns, and the class information is contained in a binary array **Y**. Each class is represented by a column in **Y**, where a value of one assigns an object to that class. **X **is logarithmically transformed prior to the analysis. No other pre-processing or scaling is performed, that is, we use the data directly as provided by Spira *et al. *[15].

The spike-in data [20] are obtained from a designed experiment where 14 groups of three transcripts each were spiked in at 14 different concentration levels in three replicates. There is thus a total of *N *= 42 objects in the experiment, corresponding to specific concentration mixtures. Expression levels were measured on a modified Affymetrix HG-U133A array, and concentrations ranging from zero to 512 pM were arranged in a Latin square setup. The 42 spike-in genes were measured in a constant human background, 22300 probe sets in total. The data are pre-processed using the gcrma-package [31], which is part of the Bioconductor project [32]. As groups of three transcripts are spiked in at the same amounts, the response matrix **Y **contains 14 concentration profiles, one for each group. Several authors have remarked that some of the background genes show consistent differential expression across the experiments [21-23]. Some of those genes contain sequences which match the spike-ins exactly. We therefore adopt the extended spike-in gene-list given by McGee and Chen [22], yielding a total of 64 entries. The list is identical to the one suggested by Lo and Gottardo [23], except the probe set "205397_x_at" is removed. This probe set has identical sequence to the originally included "205398_at" for 5 of 11 probes. Due to memory limitations, there is a need to filter away some genes before the analysis. We therefore include only those normalised probe sets with a standard deviation above 0.4, which results in a total of *M *= 14.490 variables in **X**. Potential alternatives to filtering would have been to apply a leave-two-out cross-validation, or to use fewer **Y**-variables or components in the analysis.

In the liver toxicity study [24], male rats of the inbred strain Fisher 344 were administered non-toxic (50 or 150 mg/kg), moderately toxic (1500 mg/kg) or severely toxic (2000 mg/kg) doses of paracetamol in a controlled experiment. Necropsies were performed at 6, 18, 24 and 48 hours after exposure. RNA analysis was performed on an Agilent platform (Agilent Technologies, USA), and four biological replicates were obtained for each of the 16 design-points. All spots flagged as bad or not found by the image analysis program are removed. In addition, spots are removed if they have total intensity less than 200, foreground to background intensity less than 1.5, spot diameter less then 25 pixels, or more than 50% of pixels saturated. As the background estimates available in the Agilent image analysis report are local backgrounds measured in pixels near the spots, it is doubtful if this is a useful estimate of the background in the spot [33]. Therefore, no background correction is done. The filtered data are transformed to *log*_2_-ratios and normalised using global loess to improve colour balance and remove global intensity effects [34]. Ratios for spots that have been removed in the filtering are imputed using k-nearest neighbours with *k *= 10 [35]. Two histological and ten clinical variables containing markers for liver injury are available for each object. The expression data are arranged in a matrix **X **of *N *= 64 objects and *M *= 7445 expression levels. For analysis by SAM, the two non-toxic levels are pooled in a group of low dose, and the two toxic levels are pooled in a group of high dose. The data are further divided into groups for each of the four time-points, giving a total of 8 groups to be tested for differential expression. In the bilinear analysis, a matrix **Y **of 14 columns is constructed instead that contains the design information of dose and time as well as the phenotypic responses available. A description of all the variables in **Y **are given in figure 8. The **Y**-variables SDH, BILE, AST and ALT are log-transformed in order to get responses which are more closely bell-shaped.

### Bridge-PLS regression

PLSR finds linear combinations (scores) of the original features spanning directions of high covariance between **X **and **Y**. In the most common PLSR implementation, the data are deflated each time a component is found, by subtraction of that component from the remaining **X**-data. This ensures that each score vector is orthogonal to all previous components. It also enables better model fit, as several components are allowed in the modelling of each individual response. Cross-validation is usually applied to find the number of components which yields the best predictions [3]. However, the best prediction model does not necessarily contain the most informative components. If a single response is modelled (i.e. if **Y **has only one column), qualitatively useful information is expected from the vector of covariances between the variables in **X **and the response [36,37]. The covariances are given by the so-called loading weights corresponding to the first component, and this vector is therefore well suited for significance analysis. Subsequent loading weights are less interpretable, as they, due to the deflation step, may be influenced by information in **X **unrelated to **Y**. In order to obtain informative weights for multiple responses, it is possible to make one PLSR-model for each individual response. Such an approach was taken e.g. in [9], where cell cycle regulated genes were found using the first loading weight from two single-response PLSR-models.

Bridge-PLSR has no deflation step, and the loading weights are used directly in the model rather than calculating new loadings [14]. If the ridge-parameter *γ *is set to zero, Bridge-PLSR is simply a singular value decomposition of the covariance matrix **X*'*Y**, with a subsequent prediction relation between **X **and **Y**. At the other extreme, when *γ *= 1, Bridge-PLSR becomes an ordinary principal component regression [3]. Any intermediate value of *γ *corresponds to some weighted average between those two methods. For significance analysis, and for interpretation of components in relation to **Y**, the optimal value of *γ *is zero. The loadings will then collectively explain the same **Y**-information as the set of loading weights from all one-component, individual response PLSR-models. For multiple correlated responses, Kvalheim [38] recommends orthogonalisation of **Y **before calculating such single-response models. This ensures that the models will be close to orthogonal to each other as long as the predictive relation between **X **and **Y **is good. These steps are all circumvented with Bridge-PLSR, as the Bridge-PLSR loadings are orthogonal by default. In addition, a single, predictive Bridge-PLSR model is more useful and appealing to work with than multiple single-response PLSR-models.

When *γ *= 0, the maximum number of components equals the sum of independent responses in **Y**. If all these components are used, the residuals will be orthogonal to **Y**, and the loadings (= loading weights) will together account for all the **Y**-related information in **X**. Because only one component is available for each orthogonal response when *γ *= 0, the predictive ability of Bridge-PLSR is often less than that of an ordinary PLSR. However, a tuning of the *γ*-parameter may increase the predictive ability of the model. This is expected for instance in cases where **Y **is large or noisy compared to **X**. It is also seen from the response surfaces provided in supplementary information that a larger *γ*-value may stabilise the decomposition such that early local minima are avoided. However, the response surfaces provided are not validated by an external validation and should not be used for optimisation. Any such tuning of parameters should be accompanied for instance by cross model validation to avoid optimistically biased results [19,39].

For the analysis in this work, *γ *is set to the small default value of 0.01. It is seen from the response surfaces in supplementary information that this yields the same predictive ability (down to two decimals) as if *γ *were zero. Also, the components obtained using the two values of *γ *are qualitatively equal, such that the influence on the significance analysis is negligible. The *γ*-parameter is given this value only to enable interpretation of more than two components for the smoker data, although these components are not used in the jack-knife. Bridge-PLSR is algorithmically simple, and the models are validated by cross-validation or test sets in a straight forward manner.

#### Pseudo-code Bridge-PLSR

1. Start with column-centred **X **and **Y**

2. Find by SVD **Y **= **U**_*y*_**S**_*y*_VyT
 MathType@MTEF@5@5@+=feaafiart1ev1aaatCvAUfKttLearuWrP9MDH5MBPbIqV92AaeXatLxBI9gBaebbnrfifHhDYfgasaacH8akY=wiFfYdH8Gipec8Eeeu0xXdbba9frFj0=OqFfea0dXdd9vqai=hGuQ8kuc9pgc9s8qqaq=dirpe0xb9q8qiLsFr0=vr0=vr0dc8meaabaqaciaacaGaaeqabaqabeGadaaakeaaieqacqWFwbGvdaqhaaWcbaGaeOyEaKhabaGaeOivaqfaaaaa@30CE@

3. Construct a representation of **Y **that has the rank of **X**: **G **= (1 - *γ*) **U**_*y*_**S**_*y*_UyT
 MathType@MTEF@5@5@+=feaafiart1ev1aaatCvAUfKttLearuWrP9MDH5MBPbIqV92AaeXatLxBI9gBaebbnrfifHhDYfgasaacH8akY=wiFfYdH8Gipec8Eeeu0xXdbba9frFj0=OqFfea0dXdd9vqai=hGuQ8kuc9pgc9s8qqaq=dirpe0xb9q8qiLsFr0=vr0=vr0dc8meaabaqaciaacaGaaeqabaqabeGadaaakeaaieqacqWFvbqvdaqhaaWcbaGaemyEaKhabaGaemivaqfaaaaa@30BE@ + *γ***I**

4. Find by SVD **X**^*T*^**G **= **USV**^*T*^

5. Loadings **P**_*A *_given by the *A *largest components in **U**

6. Scores **T**_*A *_= **XP**_*A*_

7. **Y**-loadings **Q**_*A *_= **Y**^*T*^**T**_*A*_(TAT
 MathType@MTEF@5@5@+=feaafiart1ev1aaatCvAUfKttLearuWrP9MDH5MBPbIqV92AaeXatLxBI9gBaebbnrfifHhDYfgasaacH8akY=wiFfYdH8Gipec8Eeeu0xXdbba9frFj0=OqFfea0dXdd9vqai=hGuQ8kuc9pgc9s8qqaq=dirpe0xb9q8qiLsFr0=vr0=vr0dc8meaabaqaciaacaGaaeqabaqabeGadaaakeaaieqacqWFubavdaqhaaWcbaGaemyqaeeabaGaemivaqfaaaaa@304C@**T**_*A*_)^-1^

8. Regression coefficients **B **= **P**_*A*_QAT
 MathType@MTEF@5@5@+=feaafiart1ev1aaatCvAUfKttLearuWrP9MDH5MBPbIqV92AaeXatLxBI9gBaebbnrfifHhDYfgasaacH8akY=wiFfYdH8Gipec8Eeeu0xXdbba9frFj0=OqFfea0dXdd9vqai=hGuQ8kuc9pgc9s8qqaq=dirpe0xb9q8qiLsFr0=vr0=vr0dc8meaabaqaciaacaGaaeqabaqabeGadaaakeaaieqacqWFrbqudaqhaaWcbaGaemyqaeeabaGaemivaqfaaaaa@3046@

9. Predictive relation Y^=1b0T+XB
 MathType@MTEF@5@5@+=feaafiart1ev1aaatCvAUfKttLearuWrP9MDH5MBPbIqV92AaeXatLxBI9gBaebbnrfifHhDYfgasaacH8akY=wiFfYdH8Gipec8Eeeu0xXdbba9frFj0=OqFfea0dXdd9vqai=hGuQ8kuc9pgc9s8qqaq=dirpe0xb9q8qiLsFr0=vr0=vr0dc8meaabaqaciaacaGaaeqabaqabeGadaaakeaaieqacuWFzbqwgaqcaiabg2da9iab=fdaXiab=jgaInaaDaaaleaacqaIWaamaeaacqWGubavaaGccqGHRaWkcqWFybawcqWFcbGqaaa@36A9@, where the offset b0=y¯−BTx¯
 MathType@MTEF@5@5@+=feaafiart1ev1aaatCvAUfKttLearuWrP9MDH5MBPbIqV92AaeXatLxBI9gBaebbnrfifHhDYfgasaacH8akY=wiFfYdH8Gipec8Eeeu0xXdbba9frFj0=OqFfea0dXdd9vqai=hGuQ8kuc9pgc9s8qqaq=dirpe0xb9q8qiLsFr0=vr0=vr0dc8meaabaqaciaacaGaaeqabaqabeGadaaakeaaieqacqWFIbGydaWgaaWcbaGaeGimaadabeaakiabg2da9iqb=Lha5zaaraGaeyOeI0Iae8Nqai0aaWbaaSqabeaacqWGubavaaGccuWF4baEgaqeaaaa@36A3@

#### Optional extensions

Even though all the **Y**-related information is accounted for in Bridge-PLSR, the model may also include variance which is orthogonal to **Y**. Such orthogonal variance is the reason the smoker-classes are not better separated in figure 4. In a perfectly fitted model, all the samples of either class would appear as a single spot in the score plot, such that the three classes formed a triangle. In general, perfect fit is not a goal, as such a model would describe also measurement noise and other artifacts. However, in cases with much irrelevant **X**-variation, removal of some unwanted structures might improve the analysis. At least three approaches can be taken to this end. Variable selection attempts to remove the least relevant variables from **X**, improving the subsequent data-analysis [19]. Target rotation of PLSR loading weights toward orthogonalised responses will improve the fit provided the number of initial components is sufficiently high [37,38]. Target rotation of two Bridge-PLSR components toward two independent responses will give no improvement, as the Bridge-PLSR loadings already span the directions of interest (when *γ *= 0). A third approach is to use some sort of orthogonal signal correction, a family of methods which separate components from **X **which are orthogonal to **Y **[40,41]. The method of 02-PLS [40] is especially interesting, as this model formulation is very similar to Bridge-PLSR. In a test using the smoker data, target rotation based on five PLSR components gave similar results as an O2-PLS model where three orthogonal components had been removed (not shown).

Removal of some unwanted **X**-information may increase the predictive ability and find even more relevant genes. However, all the above procedures for improving the modelling are based on the data themselves, which means that degrees of freedom are in effect removed from **X**. It follows that the risk of overfit increases the more the data are manipulated. As an example, a perfect fit will always result when a target rotation is based on all data, or when all orthogonal components are removed from the model. Much caution and good knowledge of the data are therefore advised when any of the above procedures are implemented. As these methods are useful only for data with much irrelevant **X**-variation, we have chosen not to include them further in this work. As was demonstrated with the smoker-data, Bridge-PLSR will find the important directions in the data no matter how much irrelevant **X**-structure is present.

### Significance analysis

A cross-validation using an appropriate dimension reduction method is performed, and loadings from each sub-model with left-out objects is obtained. The perturbed loadings are used to estimate the stability of each feature. The optimal number of components *A *for prediction, and the overall predictability of the model may be estimated as described in [3]. Only components that lead to a significant decrease in the cross-validated prediction error should then be included. In this work, we concentrate more on significance analysis than on optimisation of prediction models, and interpretation of score plots is therefore at least as important when *A *is found.

Many of the dimension reduction methods yield orthonormal loadings, in which cases the relative importance of the components are reflected by the length of the scores. For the significance analysis, it is important that the loadings themselves are weighted by the variance of their components. All perturbed loadings from the cross-validation also have to be aligned properly. A bilinear model can be written on the form X^=TAPAT=TACC−1PAT
 MathType@MTEF@5@5@+=feaafiart1ev1aaatCvAUfKttLearuWrP9MDH5MBPbIqV92AaeXatLxBI9gBaebbnrfifHhDYfgasaacH8akY=wiFfYdH8Gipec8Eeeu0xXdbba9frFj0=OqFfea0dXdd9vqai=hGuQ8kuc9pgc9s8qqaq=dirpe0xb9q8qiLsFr0=vr0=vr0dc8meaabaqaciaacaGaaeqabaqabeGadaaakeaaieqacuWFybawgaqcaiabg2da9iab=rfaunaaBaaaleaacqWGbbqqaeqaaOGae8huaa1aa0baaSqaaiabdgeabbqaaiabdsfaubaakiabg2da9iab=rfaunaaBaaaleaacqWGbbqqaeqaaOGae83qamKae83qam0aaWbaaSqabeaacqGHsislcqaIXaqmaaGccqWFqbaudaqhaaWcbaGaemyqaeeabaGaemivaqfaaaaa@4033@, where **C **is any invertible matrix of rank *A*. This means that the components have full rotational freedom, and it is often observed that a component is flipped 180° in one sub-model relative to another. Components of similar eigenvalues may also be sorted differently in different sub-models. A Procrustes rotation of the perturbed models toward the calibration model will correct for these inconsistencies [3]. We perform un-centred and un-scaled rotations of all loadings prior to significance testing.

Each variable (gene) is tested under the null hypothesis that its corresponding loading-parameter is zero in the *A*-dimensional space spanned by the components. It follows that the choice of dimension reduction is very important for the outcome of the test. Figures 1 and 2 show that components spanning only irrelevant **X**-variation will yield irrelevant genes. On the other hand, when the components describe some **Y**-information of interest, we directly find the features important for the different responses (e.g. figures 4 and 5). Hotelling's *T*^2^-test is able to assess the significance of parameters in several dimensions simultaneously. We have modified the test to make it more suited for jack-knife of bilinear model parameters. Some of these modifications are described in [3,12] for the regular one-sample t-test. The variation of the perturbed sub-models around an estimated true model describes the stability for each gene. Traditionally, the mean of the sub-models represents the centre points, but the median or even the full calibration model may also be used. Because the perturbed models are not independent of the full model, the deviations are summed instead of averaged. The latter point makes a difference if the actual value of the *T*^2^-score is interpreted, not if the *T*^2^-score is used simply as a ranking of the genes. Finally, shrinkage of the gene-specific variances toward a total variance across genes is included [25]. This is to avoid poorly estimated variances due to the small number of objects in microarray experiments. A shrinkage factor of zero means that every gene is evaluated individually, while a factor of one treats all genes as having equal variances. The actual value of the parameter is a choice that must be made on the basis of the number of replicates and the overall data quality. Poor quality data and few replicates may justify increased shrinkage.

The *T*^2^-score for gene *i *∈ {1,..., *M*} is given as

Ti2=piTSi−1pi
 MathType@MTEF@5@5@+=feaafiart1ev1aaatCvAUfKttLearuWrP9MDH5MBPbIqV92AaeXatLxBI9gBaebbnrfifHhDYfgasaacH8akY=wiFfYdH8Gipec8Eeeu0xXdbba9frFj0=OqFfea0dXdd9vqai=hGuQ8kuc9pgc9s8qqaq=dirpe0xb9q8qiLsFr0=vr0=vr0dc8meaabaqaciaacaGaaeqabaqabeGadaaakeaacqWGubavdaqhaaWcbaGaemyAaKgabaGaeGOmaidaaOGaeyypa0dcbeGae8hCaa3aa0baaSqaaiabdMgaPbqaaiabdsfaubaakiab=nfatnaaDaaaleaacqWGPbqAaeaacqGHsislcqaIXaqmaaGccqWFWbaCdaWgaaWcbaGaemyAaKgabeaaaaa@3D1F@

Si=(1−β)⋅Sigene+β⋅Stot
 MathType@MTEF@5@5@+=feaafiart1ev1aaatCvAUfKttLearuWrP9MDH5MBPbIqV92AaeXatLxBI9gBaebbnrfifHhDYfgasaacH8akY=wiFfYdH8Gipec8Eeeu0xXdbba9frFj0=OqFfea0dXdd9vqai=hGuQ8kuc9pgc9s8qqaq=dirpe0xb9q8qiLsFr0=vr0=vr0dc8meaabaqaciaacaGaaeqabaqabeGadaaakeaaieqacqWFtbWudaWgaaWcbaGaemyAaKgabeaakiabg2da9maabmaabaGaeGymaeJaeyOeI0ccciGae4NSdigacaGLOaGaayzkaaGaeyyXICTae83uam1aa0baaSqaaiabdMgaPbqaaiabdEgaNjabdwgaLjabd6gaUjabdwgaLbaakiabgUcaRiab+j7aIjabgwSixlab=nfatnaaCaaaleqabaGaemiDaqNaem4Ba8MaemiDaqhaaaaa@4A56@

Sigene=(PiCV−1piT)T(PiCV−1piT)⋅g
 MathType@MTEF@5@5@+=feaafiart1ev1aaatCvAUfKttLearuWrP9MDH5MBPbIqV92AaeXatLxBI9gBaebbnrfifHhDYfgasaacH8akY=wiFfYdH8Gipec8Eeeu0xXdbba9frFj0=OqFfea0dXdd9vqai=hGuQ8kuc9pgc9s8qqaq=dirpe0xb9q8qiLsFr0=vr0=vr0dc8meaabaqaciaacaGaaeqabaqabeGadaaakeaaieqacqWFtbWudaqhaaWcbaGaemyAaKgabaGaem4zaCMaemyzauMaemOBa4MaemyzaugaaOGaeyypa0JaeiikaGIae8huaa1aa0baaSqaaiabdMgaPbqaaiabdoeadjabdAfawbaakiabgkHiTiab=fdaXiab=bhaWnaaDaaaleaacqWGPbqAaeaacqWGubavaaGccqGGPaqkdaahaaWcbeqaaiabdsfaubaakiabcIcaOiab=bfaqnaaDaaaleaacqWGPbqAaeaacqWGdbWqcqWGwbGvaaGccqGHsislcqWFXaqmcqWFWbaCdaqhaaWcbaGaemyAaKgabaGaemivaqfaaOGaeiykaKIaeyyXICTaem4zaCgaaa@5436@

The vector **p**_*i *_is of length *A *and represents the true loadings, *β *∈ [0, 1] is the shrinkage factor, PiCV
 MathType@MTEF@5@5@+=feaafiart1ev1aaatCvAUfKttLearuWrP9MDH5MBPbIqV92AaeXatLxBI9gBaebbnrfifHhDYfgasaacH8akY=wiFfYdH8Gipec8Eeeu0xXdbba9frFj0=OqFfea0dXdd9vqai=hGuQ8kuc9pgc9s8qqaq=dirpe0xb9q8qiLsFr0=vr0=vr0dc8meaabaqaciaacaGaaeqabaqabeGadaaakeaaieqacqWFqbaudaqhaaWcbaGaemyAaKgabaGaem4qamKaemOvayfaaaaa@31A7@ is a matrix of size *K*-by-*A *holding the loadings from the *K *sub-models and **1 **is a vector of ones. **S**^*tot *^is the mean or median of the scaled, gene-specific variance-covariance matrices Sigene
 MathType@MTEF@5@5@+=feaafiart1ev1aaatCvAUfKttLearuWrP9MDH5MBPbIqV92AaeXatLxBI9gBaebbnrfifHhDYfgasaacH8akY=wiFfYdH8Gipec8Eeeu0xXdbba9frFj0=OqFfea0dXdd9vqai=hGuQ8kuc9pgc9s8qqaq=dirpe0xb9q8qiLsFr0=vr0=vr0dc8meaabaqaciaacaGaaeqabaqabeGadaaakeaaieqacqWFtbWudaqhaaWcbaGaemyAaKgabaGaem4zaCMaemyzauMaemOBa4Maemyzaugaaaaa@34CB@ across all genes. The scaling g=K−1K
 MathType@MTEF@5@5@+=feaafiart1ev1aaatCvAUfKttLearuWrP9MDH5MBPbIqV92AaeXatLxBI9gBaebbnrfifHhDYfgasaacH8akY=wiFfYdH8Gipec8Eeeu0xXdbba9frFj0=OqFfea0dXdd9vqai=hGuQ8kuc9pgc9s8qqaq=dirpe0xb9q8qiLsFr0=vr0=vr0dc8meaabaqaciaacaGaaeqabaqabeGadaaakeaacqWGNbWzcqGH9aqpdaWcaaqaaiabdUealjabgkHiTiabigdaXaqaaiabdUealbaaaaa@3334@ is taken from [3] and is close to one in most cases. In this work, **p**_*i *_is the mean value over sub-models, the coefficient *β *= 0.1 and the median of covariances is used to find **S**^*tot*^.

### FDR estimation

We adapt the definition of the positive false discovery rate [42], which states that

FDR=E[FPFP+TP|(FP+TP)>0]
 MathType@MTEF@5@5@+=feaafiart1ev1aaatCvAUfKttLearuWrP9MDH5MBPbIqV92AaeXatLxBI9gBaebbnrfifHhDYfgasaacH8akY=wiFfYdH8Gipec8Eeeu0xXdbba9frFj0=OqFfea0dXdd9vqai=hGuQ8kuc9pgc9s8qqaq=dirpe0xb9q8qiLsFr0=vr0=vr0dc8meaabaqaciaacaGaaeqabaqabeGadaaakeaacqqGgbGrcqqGebarcqqGsbGucqGH9aqpcqqGfbqrdaWadaqaamaaeiaabaWaaSaaaeaacqWGgbGrcqWGqbauaeaacqWGgbGrcqWGqbaucqGHRaWkcqWGubavcqWGqbauaaaacaGLiWoacqGGOaakcqWGgbGrcqWGqbaucqGHRaWkcqWGubavcqWGqbaucqGGPaqkcqGH+aGpcqaIWaamaiaawUfacaGLDbaaaaa@4682@

*FP *and *TP *are the numbers of false and true positives, respectively. A q-value ∈ [0, 1] assigned to each gene gives the minimum FDR that results in a rejected hypothesis for that feature. An FDR significance level cutoff is applied to the q-values to decide which features to call significant. The conditional statement that the sum of positive outcomes must be larger than zero can often be disregarded in practice. It is then possible to estimate the q-values by resampling, using the relation

q^i=FP^i(FP+TP)i
 MathType@MTEF@5@5@+=feaafiart1ev1aaatCvAUfKttLearuWrP9MDH5MBPbIqV92AaeXatLxBI9gBaebbnrfifHhDYfgasaacH8akY=wiFfYdH8Gipec8Eeeu0xXdbba9frFj0=OqFfea0dXdd9vqai=hGuQ8kuc9pgc9s8qqaq=dirpe0xb9q8qiLsFr0=vr0=vr0dc8meaabaqaciaacaGaaeqabaqabeGadaaakeaacuWGXbqCgaqcamaaBaaaleaacqWGPbqAaeqaaOGaeyypa0ZaaSaaaeaadaqiaaqaaiabdAeagjabdcfaqbGaayPadaWaaSbaaSqaaiabdMgaPbqabaaakeaacqGGOaakcqWGgbGrcqWGqbaucqGHRaWkcqWGubavcqWGqbaucqGGPaqkdaWgaaWcbaGaemyAaKgabeaaaaaaaa@3E12@

Every feature *i *is associated with a score Ti2
 MathType@MTEF@5@5@+=feaafiart1ev1aaatCvAUfKttLearuWrP9MDH5MBPbIqV92AaeXatLxBI9gBaebbnrfifHhDYfgasaacH8akY=wiFfYdH8Gipec8Eeeu0xXdbba9frFj0=OqFfea0dXdd9vqai=hGuQ8kuc9pgc9s8qqaq=dirpe0xb9q8qiLsFr0=vr0=vr0dc8meaabaqaciaacaGaaeqabaqabeGadaaakeaacqWGubavdaqhaaWcbaGaemyAaKgabaGaeGOmaidaaaaa@3057@. The denominator holds the total number of genes at least as significant as this based on the calibration model. The number of false positives at the same significance level is not known, and must be estimated. The estimated count for gene *i *is found by reshuffling and denoted FP^
 MathType@MTEF@5@5@+=feaafiart1ev1aaatCvAUfKttLearuWrP9MDH5MBPbIqV92AaeXatLxBI9gBaebbnrfifHhDYfgasaacH8akY=wiFfYdH8Gipec8Eeeu0xXdbba9frFj0=OqFfea0dXdd9vqai=hGuQ8kuc9pgc9s8qqaq=dirpe0xb9q8qiLsFr0=vr0=vr0dc8meaabaqaciaacaGaaeqabaqabeGadaaakeaadaqiaaqaaiabdAeagjabdcfaqbGaayPadaaaaa@2FAC@_*i*_. The rows of **Y **are shuffled *R *times to give several matrices Yr∗
 MathType@MTEF@5@5@+=feaafiart1ev1aaatCvAUfKttLearuWrP9MDH5MBPbIqV92AaeXatLxBI9gBaebbnrfifHhDYfgasaacH8akY=wiFfYdH8Gipec8Eeeu0xXdbba9frFj0=OqFfea0dXdd9vqai=hGuQ8kuc9pgc9s8qqaq=dirpe0xb9q8qiLsFr0=vr0=vr0dc8meaabaqaciaacaGaaeqabaqabeGadaaakeaaieqacqWFzbqwdaqhaaWcbaGaemOCaihabaGaey4fIOcaaaaa@3076@, *r *∈ {1,..., *R*}, with random object-information only. A jack-knife based on one of the randomised matrices is expected to give no true positives, especially if the number of significant features is small compared to the total number of features tested. Here, FP^
 MathType@MTEF@5@5@+=feaafiart1ev1aaatCvAUfKttLearuWrP9MDH5MBPbIqV92AaeXatLxBI9gBaebbnrfifHhDYfgasaacH8akY=wiFfYdH8Gipec8Eeeu0xXdbba9frFj0=OqFfea0dXdd9vqai=hGuQ8kuc9pgc9s8qqaq=dirpe0xb9q8qiLsFr0=vr0=vr0dc8meaabaqaciaacaGaaeqabaqabeGadaaakeaadaqiaaqaaiabdAeagjabdcfaqbGaayPadaaaaa@2FAC@ is found as the median number of significant outcomes over *R *= 1000 shuffled responses, and all features with q^i
 MathType@MTEF@5@5@+=feaafiart1ev1aaatCvAUfKttLearuWrP9MDH5MBPbIqV92AaeXatLxBI9gBaebbnrfifHhDYfgasaacH8akY=wiFfYdH8Gipec8Eeeu0xXdbba9frFj0=OqFfea0dXdd9vqai=hGuQ8kuc9pgc9s8qqaq=dirpe0xb9q8qiLsFr0=vr0=vr0dc8meaabaqaciaacaGaaeqabaqabeGadaaakeaacuWGXbqCgaqcamaaBaaaleaacqWGPbqAaeqaaaaa@2FAE@ smaller than a pre-set significance level *α *are called significant. Estimation of FP^
 MathType@MTEF@5@5@+=feaafiart1ev1aaatCvAUfKttLearuWrP9MDH5MBPbIqV92AaeXatLxBI9gBaebbnrfifHhDYfgasaacH8akY=wiFfYdH8Gipec8Eeeu0xXdbba9frFj0=OqFfea0dXdd9vqai=hGuQ8kuc9pgc9s8qqaq=dirpe0xb9q8qiLsFr0=vr0=vr0dc8meaabaqaciaacaGaaeqabaqabeGadaaakeaadaqiaaqaaiabdAeagjabdcfaqbGaayPadaaaaa@2FAC@ when no **Y **is available is difficult, because it is not possible to shuffle **X **for each feature separately without simultaneously disrupting all other systematic structure in the data. For the smoker-data, we have therefore used the same number of significant features for PCA as we found for Bridge-PLSR. The FDR significance level is set to *α *= 0.05 for the smoker-data and to *α *= 0.01 for the spike-in data. Because the relevant signal in the paracetamol data set is very strong, a significance level of *α *= 0.001 is enough to get a large number of significant features for these data.

### SAM and Limma analysis

SAM and Limma are used to identify differentially expressed genes in the smoker data set. Both methods are part of the Bioconductor software (Version 1.9) [32], and we use the packages limma and siggenes for Limma and SAM, respectively. Limma is performed with the functions lmFit and eBayes with default settings. We control Benjamini and Hochberg's FDR at a level of *α *using the function decideTests. SAM is run with the function sam.dstat with 1000 permutations, the parameter *delta *= 0.8 and the remaining parameters at default setting.

## Competing interests

The author(s) declares that there are no competing interests.

## Authors' contributions

LG wrote the manuscript. LG, EA and AF contributed equally to method development, programming and data analysis. BKA (group leader) helped to draft the manuscript. All authors read and approved the final version.

## Supplementary Material

Additional file 1 **Matlab toolbox**. This is a zip-compressed archive containing the Matlab functions used in the work. The files are published under a GNU General Public License, and an example-script and a small data set for demonstration are included. The code is written in a Linux operating system, but the Matlab-functions are tested also in Windows. The archive contains the following files:
BRIDGE_PLSR/extendedCV.m
BRIDGE_PLSR/estimateFDR.m
BRIDGE_PLSR/pcaExt.m
BRIDGE_PLSR/bridgeExt.m
BRIDGE_PLSR/tSquare.m
BRIDGE_PLSR/procrust.m
BRIDGE_PLSR/bridgeplsr.m
BRIDGE_PLSR/plsrExt.m
BRIDGE_PLSR/multTest.m
BRIDGE_PLSR/aOptFind.m
BRIDGE_PLSR/pca.m
BRIDGE_PLSR/plsrConst.m
BRIDGE_PLSR/bilinearSign.m
BRIDGE_PLSR/LICENSE
BRIDGE_PLSR/README
BRIDGE_PLSR/example _script.m
BRIDGE_PLSR/plotDiagnosis.m
BRIDGE_PLSR/plotScatter.m
BRIDGE_PLSR/plotDistributionP.m
BRIDGE_PLSR/data/readme.txt
BRIDGE_PLSR/data/example_data.mat Click here for file

Additional file 2 **Annotations for genes uniquely detected by Bridge-PLSR. **This is a zip-compressed archive containing a Microsoft Excel spreadsheet with the list of genes (plsOnlyAnnot.xls), and a description file (README.txt) Click here for file

Additional file 3 **Response surfaces for optimisation of *γ *and *A***. This file is formatted in portable document format. Click here for file
