# Supplementary material for: A framework for significance analysis of gene expression data using dimension reduction methods
Source: BMC Bioinformatics. 2007 Sep 18;8:346. doi: 10.1186/1471-2105-8-346 (PMC2194745; doi:10.1186/1471-2105-8-346)
Supplement: Additional file 3 — Response surfaces for optimisation of γ and A. This file is formatted in portable document format. [file 1471-2105-8-346-S3.pdf]

Response surfaces for optimisation of γ and A

The explained **Y**-variances for the data sets used in the manuscript, as estimated by a leave-one-out cross-validation. Results for both Bridge-PLSR and ordinary PLSR are given. Bridge-PLSR models are optimised for several values of the ridge-parameter γ, where $\gamma = 1$ corresponds to an ordinary PCA decomposition. The optimal number of components A for prediction is estimated by requiring an improvement of two standard errors for each included component. The resulting model rank is highlighted for each model. As the optimisation of parameters was not included in an external validation, some of these results may be over-optimistic. However, as the main goal in this work was significance analysis, the optimal models for prediction was not used. The precision (the number of significant digits) of the variance estimates is arbitrary and does not reflect the uncertainty of the results.

Table 1: The explained variance for the smoker-data. The response surface is robust with regard to γ for small values of this parameter. Many of the Bridge-PLSR models and the PLSR model reaches a local minimum in the prediction error after one or two components

Bridge-PLSR (% explained variance)						
γ	$A=1$	$A=2$	$A=3$	$A=4$	$A=5$	$A=6$
0	**20.3473**	17.4917	-	-	-	-
0.01	**20.3477**	17.4915	25.9869	35.6092	38.8020	38.6147
0.1	**20.3349**	17.4633	25.9750	35.6020	38.7984	38.6111
0.2	**20.2701**	17.3553	25.9301	35.5745	38.7848	38.5975
0.3	**20.1174**	17.1165	25.8347	35.5138	38.7547	38.5673
0.4	**19.8115**	16.6538	25.6677	35.3969	38.6971	38.5094
0.5	**19.2248**	15.7845	25.4276	35.1798	38.5906	38.4024
0.6	**18.0835**	14.1127	25.2874	34.7726	38.3916	38.2025
0.7	15.7390	18.9894	26.3544	33.9848	**37.9975**	37.8071
0.8	10.6827	18.2614	28.5223	32.7304	**37.1067**	36.9147
0.9	3.1957	7.5559	28.1377	31.1243	**34.4112**	34.2457
1	0.5503	0.9204	12.1192	28.6304	29.1400	**29.4811**

PLSR (% explained variance)						
	20.3472	**32.3844**	29.3989	36.8339	42.6600	42.2927

Table 2: The explained variances for the rat-liver data. For these data, there is not much difference between the bilinear models.

Bridge-PLSR (% explained variance)						
γ	$A=1$	$A=2$	$A=3$	$A=4$	$A=5$	$A=6$
0	28.4094	41.7311	48.5116	**53.1165**	53.7909	53.1411
0.01	28.4094	41.7298	48.5093	**53.1162**	53.7909	53.1440
0.1	28.4097	41.7153	48.4828	**53.1126**	53.7902	53.1756
0.2	28.4101	41.6945	48.4423	**53.1071**	53.7890	53.2187
0.3	28.4106	41.6662	48.3838	**53.0993**	53.7870	53.2753
0.4	28.4113	41.6260	48.2949	**53.0878**	53.7836	53.3551
0.5	28.4123	41.5652	48.1506	**53.0698**	53.7776	53.4820
0.6	28.4137	41.4647	47.8924	**53.0399**	53.7663	53.7642
0.7	28.4160	41.2754	47.3511	**52.9856**	53.7414	53.9482
0.8	28.4205	40.8262	45.2238	52.8729	**53.6647**	54.0464
0.9	28.4322	39.0808	43.1108	52.5767	52.5876	**54.1928**
1	28.5198	29.4049	44.0042	**51.0474**	50.4910	49.2953

PLSR (% explained variance)						
	28.4094	41.7233	42.5152	**53.0019**	53.7633	48.3394
